# Calpain-Mediated Processing of Adenylate Cyclase Toxin Generates a Cytosolic Soluble Catalytically Active N-Terminal Domain

**DOI:** 10.1371/journal.pone.0067648

**Published:** 2013-06-26

**Authors:** Kepa B. Uribe, Aitor Etxebarria, César Martín, Helena Ostolaza

**Affiliations:** Unidad de Biofísica (CSIC, UPV/EHU) and Departamento de Bioquímica, Universidad del País Vasco UPV/EHU, Bilbao, Spain; University of Helsinki, Finland

## Abstract

*Bordetella pertussis*, the whooping cough pathogen, secretes several virulence factors among which adenylate cyclase toxin (ACT) is essential for establishment of the disease in the respiratory tract. ACT weakens host defenses by suppressing important bactericidal activities of the phagocytic cells. Up to now, it was believed that cell intoxication by ACT was a consequence of the accumulation of abnormally high levels of cAMP, generated exclusively beneath the host plasma membrane by the toxin N-terminal catalytic adenylate cyclase (AC) domain, upon its direct translocation across the lipid bilayer. Here we show that host calpain, a calcium-dependent Cys-protease, is activated into the phagocytes by a toxin-triggered calcium rise, resulting in the proteolytic cleavage of the toxin N-terminal domain that releases a catalytically active “soluble AC”. The calpain-mediated ACT processing allows trafficking of the “soluble AC” domain into subcellular organella. At least two strategic advantages arise from this singular toxin cleavage, enhancing the specificity of action, and simultaneously preventing an indiscriminate activation of cAMP effectors throughout the cell. The present study provides novel insights into the toxin mechanism of action, as the calpain-mediated toxin processing would confer ACT the capacity for a space- and time-coordinated production of different cAMP “pools”, which would play different roles in the cell pathophysiology.

## Introduction

*Bordetella pertussis*, the bacterium causative of whooping cough, is one of the important pathogens among infants and toddlers because despite the high vaccine coverage throughout the world, the bacterium is still circulating and killing newborns in many countries [1], [2]. The adenylate cyclase toxin (ACT) is essential in the pathogenesis of *B. pertussis* [3]–[6].

ACT, a single polypeptide of 1706 aminoacid residues (≈ 200 kDa), is a member of the RTX (Repeats-in-ToXin) family of proteins that share a characteristic calcium-binding motif of Gly- and Asp-rich nonapeptide repeats at the C-terminal end [7]–[9]. It is secreted by a dedicated Type I secretion machinery [10], [11] and post-translationally fatty acylated on conserved Lys residues [12]. Both fatty acylation and Ca^2+^-binding to the RTX domain are absolute requirements for the toxin biological activity [7]–[9]. The N-terminal part of the protein contains the catalytic adenylate cyclase domain (AC domain) (≈residues 1–400), whereas the C-terminal domain (RTX domain) (≈last 1300 residues) mediates its binding to the target cell membrane and delivery of the catalytic moiety into the interior of intact target cells, by a unique mechanism of direct translocation across the plasma membrane [7]–[9]. After membrane translocation, the catalytic domain is activated by calmodulin resulting in a 1000-fold increase in its enzymatic activity and concomitant production of abnormally high levels of cAMP from cellular ATP [13], [14]. This process is referred to as intoxication. ACT can also form cation-selective pores independently of translocation, which permeabilize cell membranes at high toxin concentrations thereby perturbing ion homeostasis [15]. In addition, it has been described that ACT triggers an elevation of intracellular Ca^2+^ through cAMP-dependent L-type-like Ca^2+^ channels [16] and through a toxin “intermediate” which transiently forms a Ca^2+^-conducting path across the cell membrane [17].

It is believed that ACT assists infection by potently suppressing the host immune response [3], [6]. The toxin invades myeloid cells such as macrophages and neutrophils and also T lymphocytes through high-affinity binding to β2 integrin molecules expressed on the surface of these immune cells that act as toxin receptors [18], [19]. In addition, ACT can also invade other cell types, such as erythrocytes, or epithelial cells, through receptor-independent binding to the plasma membrane and translocation of the catalytic domain across the lipid bilayer [7].

The toxin suppresses proinflammatory and bactericidal functions of macrophages such as chemotaxis, the oxidative burst and phagocytosis, eventually inducing cell death by apoptosis [3], [20]–[23], and it also delays the development of a systemic immune response by suppressing T cell activation [23] or favoring the differentiation of CD4^+^ T cells to the Th2 and Th17 lineages and concomitant impairment in Th1 cell development [24]. Other toxin effects more recently reported include the induction of cell cycle arrest [25] or the disengagement of the LFA-1 integrin from the immune synapse [19]. These cytotoxic effects of ACT on the cells have been largely ascribed to the production of high levels of cAMP by the toxin, though non cAMP-mediated effects, such as depletion of cellular ATP, are also important in the actions of ACT [7], [26]–[28].

Intoxication of cells by ACT is the final result of several, yet not fully understood, consecutive steps: binding to the integrin receptor CD11b/CD18, insertion of the RTX domain into the lipid bilayer, direct translocation of the N-terminal catalytic domain across the lipid bilayer, binding of cellular calmodulin to the AC domain and cAMP generation [7], [9]. One of the less investigated issues in the toxin mechanism of action is the fate of the AC domain inside the host cell. Here we provide evidence that upon translocation the toxin N-terminal domain is cleaved off into macrophages by the cellular calpains, releasing a catalytically active “soluble” AC domain, which migrates to subcellular organelles.

## Materials and Methods

### Antibodies and Reagents

Anti-adenylate cyclase toxin RTX domain mouse monoclonal antibody (MAb 9D4) and anti-adenylate cyclase toxin AC domain mouse monoclonal antibody (MAb 3D1), anti-adenylate cyclase toxin AC domain (b-300) rabbit polyclonal antibody, calpain substrate IV cell permeable, m-calpain siRNA and control siRNA were from Santa Cruz Biotechnology (Santa Cruz, CA, USA); anti-nucleoporin p62 mouse monoclonal antibody was from BD Biosciences (USA); KH7, nifedipine and N-ethylmaleimide (NEM) were from Sigma-Aldrich (St Louis, MI); anti-calregulin, anti-VDAC/porin and anti-histone H2B were from abcam (Cambridge, UK); calpeptin, m-calpain (calpain 2) and calpain inhibitor type VI (SJA6017) were from Calbiochem (Merk, Germany); anti-mouse FITC, Hoechst and Mitotracker were from Invitrogen, Molecular Probes (Carlsbad, CA).

### ACT Purification

ACT was expressed in *Escherichia coli* XL-1 Blue cells (Stratagene) transformed with pT7CACT1 plasmid, kindly provided by Dr. Peter Sebo (Institute of Microbiology of the ASCR, v.v.i., Prague, Czech Republic) and purified as previously described [29].

### Isolation of Cell Membranes and Cytosol from ACT-treated Cells

J774A.1 macrophages were grown on T75 tissue culture flasks to 70–80% confluence in D-MEM culture medium containing 10% FBS and antibiotic solution (0.1 mg/ml streptomycin, 100 U/ml penicillin (Sigma, St. Louis, MO)). One hour before treatment serum was removed from the medium, and then cells were incubated with freshly purified ACT (5–35 nM) for different times (0–30 min). After that, cells were scraped and collected by centrifugation, washed twice with cold PBS supplemented with 5 mM EDTA and lysed at 4°C for 30 min in a hypotonic lysis buffer (20 mM Tris-HCl, pH 7.5, 5 mM MgCl_2_, 5 mM CaCl_2_, 1 mM DTT, 1 mM EDTA), supplemented with protease inhibitor cocktail tablets (Complete EDTA-free, Roche). For further homogenization, each sample was passed five to ten times trough a 25G needle. Then, the homogenates were centrifuged at 600 × *g* for 20 min to remove the nuclear fraction and unbroken cells, and the supernatant was ultra-centrifuged at 165,000 × *g* for 2 h. The supernatant obtained was considered the cytosolic fraction and the pellet the microsomal fraction. Protein detection was assayed by Western blot and stained with the MAb 3D1 for cytosolic and microsomal fractions and with the MAb 9D4 for microsomal fractions.

### Assay of Cytotoxicity

Cell viability of J774A.1 cells incubated with 35 nM ACT for 10 min with or without inhibitors was determined by the lactate dehydrogenase (LDH) release assay as described elsewhere [25], using the LDH Cytotoxicity assay kit (Innoprot, Spain). % Cytotoxicity = (Experimental - Blank)/Control - Blank)×100. Under these short time incubation conditions, there is no cell death.

### Measurement of Intracellular cAMP

Approximately 24 h before the start of experiments, J774A.1 cells were plated at 30,000 to 40,000 per well in 96-well tissue culture plates. Immediately before experiments, the growth medium was removed and replaced with Opti-MEM® (Invitrogen) supplemented with calcium; ACT was added directly to cells and incubated for 10 min at 37°C. Cells were washed and lysed, and cAMP measured by the direct cAMP EIA kit (Enzo lifesciences) according to the manufacturer instructions and as previously described [16]. Cell protein was measured, and data expressed as pmol cAMP/mg cell protein. Under these time and temperature conditions, there is not cell death. P values were calculated by Student's two-tailed t test.

### Measurement of the Cyclase Activity

Cyclase activity of purified fragments derived from the *in vitro* cleavage by calpain was assayed by incubation of samples (1 nM) for 10 min at 37°C with 2 nM CaM in AC reaction buffer (30 mM Tris-HCl, pH 7.4, 20 mM MgCl_2_ and 100 µM CaCl_2_), then the reaction was started by addition of 5 mM ATP. After 10 min at 37°C with continuous stirring, the reaction was stopped with 0.1 M HCl. When indicated, HCO_3_^-^ or KH7 were added to activate or inhibit cyclase activity, respectively. The cAMP production was calculated by the direct cAMP EIA kit (Enzo lifesciences).

Adenylate cyclase activity was also measured in the mitochondrial fraction and in the nuclear extracts obtained after 35 nM ACT treatment (non-treated cells were used as control). 1 µg nuclear or mitochondrial preparations were added to AC reaction buffer and the cAMP production was determined as described above.

### Measurement of Catalytic Domain Translocation and Cyclase Activity Determination in Membrane and Cytosol Fractions

To determine the translocation of the catalytic domain in presence or absence of several inhibitors (calpain inhibitors (calpeptin or SJA6017) and the L-type calcium channel inhibitor (nifedipine)), the protocol used by Eby *et al.* [28] was followed. Briefly, cells were grown on T75 tissue culture flasks, one hour before treatment serum was removed from the medium, and then cells, which had been preincubated for 30 min with the inhibitors, were incubated with freshly purified ACT (35 nM) for 10 min. The cells were then washed extensively at 4°C, and before the lysis buffer was added, half of each sample was separated and treated with 500 µg/mL trypsin (0.05% Trypsin-EDTA, Gibco) for 10 min at 4°C and the reaction stopped with 1 mM PMSF. Cell lysates were obtained disrupting the cells by osmotic shock with hypotonic buffer and cyclase activity was measured in trypsinized and non-trypsinized samples as described above. The percentage of translocation of the catalytic domain was expressed as follows: 100×(pmol cAMP produced by trypsinized cell lysates/pmol cAMP produced by non-trypsinized cell lysates). Non-trypsinized samples reflect total cell-associated ACT, while trypsinized cells reflect only the translocated, and thus trypsin-protected ACT.

The enzymatic AC activity was also determined in membrane and cytosol fractions purified from the trypsinized ACT-treated cells as described above. Levels of significance were determined by a two-tailed Student's t-test, and a confidence level of greater than 95% (p<0.05) was used to establish statistical significance.

### *In vitro* Cleavage Assay

Purified ACT and m-calpain were used in the proteolytic assay performed *in vitro*. Briefly, ACT (5 µM) was incubated with m-calpain (50 nM) in 30 mM Tris-HCl, pH 7.5, 1 mM CaCl_2_, 1.5 mM DTT buffer for 10 min at 37°C (50 µl, final volume). When required, calmodulin (10 µM) was included in the incubation buffer. In the inhibition assay, calpeptin (10 µM) was added to the incubation mixture. Reactions were terminated by boiling the samples for 10 min in SDS-PAGE loading buffer. Samples were separated by SDS-PAGE (8.5% polyacrylamide), and proteolytic processing was assessed by Western blot using an anti-AC domain mouse monoclonal antibody, MAb 3D1 and an anti-RTX domain mouse monoclonal antibody, MAb 9D4.

### Purification of the Fragments Derived from the in vitro Proteolytic Cleavage

Protein fragments were separated by molecular exclusion chromatography in a Superdex 200 10/300 GL high performance column (GE healthcare). Aliquots (0.5 ml) were collected and reaction products detected by Western blot analysis using an anti-AC domain mouse MAb 3D1. Aliquots of interest were stored at −70°C for subsequent Mass Spectrometry analysis and adenylate cyclase activity determination. Determination of the catalytic activity of the purified fragments was performed as described in the section *Measurement of cyclase activity.*

### Fluorimetric Calpain Activity Assay using a Cell Permeable FRET-based Substrate

J774A.1 cells (1×10^6^) grown in 6-well culture plates were incubated with the FRET-based fluorogenic substrate (DABCYL)-TPLK-SPPPSPRE(EDANS)RRRRRRR-NH_2_ (20 µM) for 1h at 37°C as described in [30]. Then cells were washed three times with PBS and detached, by gently pipetting up and down in 1 ml Opti-MEM® supplemented with calcium. For calpain activity measurements, cells were placed directly into 1 ml quartz cuvettes. Kinetic analysis was carried out in a Fluoromax-3 fluorimeter, at 37°C under constant stirring. The excitation was set at 320 nm, and emission monitored at 480 nm.

### Knock-down of M-calpain

J774A.1 cells were transfected with a pool of three to five target-specific 19–25 nt small interfering RNAs (siRNA) to inhibit expression of m-calpain. Protein knock-down was performed as directed by the manufacturers. Briefly, 2–5×10^5^ cells were incubated with 100 nmol of siRNA using Lipofectamine RNAiMAX reagent (Invitrogen) as carrier. 24 h post-transfection, cells were transferred to new plates and further incubated for at least 24 h before ACT treatment to determine AC domain cleavage or without treatment to assay m-calpain activity as indicator of siRNA interference effectiveness. Negative control experiments were carried out with a scrambled siRNA.

### Calpain Activity Assay

Analysis of m-calpain activity was performed taking into account the specific protease activity for α-casein in an assay developed in the laboratory. Briefly, α-casein (from bovine milk, Sigma-Aldrich) was labelled following the manufactureŕs protocol with FITC (isomer I, Sigma-Aldrich) at 1∶5 protein/fluorophore ratio. Protease activity was determined by quantification of the fluorescence intensity of the band corresponding to α-casein-FITC separated by SDS-PAGE electrophoresis. Briefly, cells (2–5×10^5^ cells) were grown and transfected with the appropiate siRNA (scrambled control or anti-m-calpain siRNA). Before the assay, control and siRNA interfered cells were incubated with 35 nM ACT for 10 min and after incubation, cells were washed twice with cold PBS supplemented with 5 mM EDTA and resuspended in 50 mM Tris-HCl, pH 7.3, supplemented with 5 mM DTT, 1 mM CaCl_2_ and 0.125 mg/mL α-casein-FITC. Then, cells were lysed and incubated for 30 minutes at 30°C to allow m-calpain activity. After that, the same volume of each sample was run in SDS-PAGE electrophoresis and developed in a ChemiDoc EQ System (Bio-Rad, USA). The intensity of the bands was measured as absence of black and related to the negative control. As negative control, m-calpain activity was also assayed in cell lysates without calcium, the intensity of the obtained band being similar to that of the α-casein alone. Levels of significance were determined by a two-tailed Student's t-test, and a confidence level of greater than 95% (p<0.05) was used to establish statistical significance.

### Confocal Microscopy

Cells were grown to sub-confluency onto 12 mm diameter glass coverslips placed into the wells of a 24-well plate. Labelling of active mitochondria was performed by incubation of cells for 15 min with Mitotracker® (500 nM). Cells were then washed three times to remove the marker in excess. ACT (35 nM) was then added and the cells were incubated for 10 min at 37°C. Then, treated cells were washed three times in cold PBS to remove unbound toxin, fixed for 10 min with 3.7% paraformaldehyde and permeabilized with cold acetone. Control cells followed the same procedure. Then samples were incubated with the anti-AC domain antibodies overnight at 4°C followed by incubation with FITC-conjugated secondary antibody. Cells were then incubated for 10 min with Hoechst to visualize nuclei. Coverslips were mounted on a glass slide and samples were visualized using a confocal microscope (Olympus IX 81) with sequential excitation and capture image acquisition with a digital camera (Axiocam NRc5, Zeiss). Images were processed with Fluoview v.50 software.

### Isolation of Mitochondria

J774A.1 cells were cultured on T75 culture flasks, as described above, 35 nM ACT was added and incubated with the cells at 37°C for 10 min. Cells were then washed three times with cold PBS, scraped and pelleted by centrifugation. All preparations were done at 4°C. The cells were resuspended in MSH-E (210 mM manitol, 70 mM sucrose, 10 mM HEPES, pH 7.4, 1 mM EDTA, supplemented with protease inhibitor cocktail tablets). Immediately afterwards the cells were homogenized using a motor-driven Teflon pestle and the homogenate was centrifuged at 1,500 × *g*, 10 min at 4°C. The obtained supernatant was recentrifuged at 7,000 × *g*, 10 min at 4°C, and the mitochondrial pellet was resuspended in MSH-E and washed twice by centrifugation (7,000 × g, 10 min at 4°C). Then, the sample was divided into aliquots and stored at −80°C.

### Isolation of Endoplasmic Reticulum

Isolation of ER of control and ACT-treated J774A.1 cells (35 nM, 10 min) was performed using standard homogenization, and differential centrifugation methods. Cells were spun at 300 × g and resuspended in 0.3 ml of homogenization buffer (8.5 mM Tris, pH 7.4, 210 mM sucrose, 8.5 mM triethanolamine, 1 mM EDTA, 4.25 mM KCl, 110 mM NaCl) plus protease inhibitors, followed by homogenization at 4°C by 12 passages through a 25G needle. Supernatants were loaded on pre-formed 0–26% Optiprep (Iodixanol, Sigma) linear gradients that had been pre-cooled to 4°C. Gradients were spun for 115 min at 280,000 × g at 4°C. Twenty fractions (600 µl each) were recovered and were subjected to electrophoresis and immunoblotting to detect the fraction corresponding to ER for further analysis.

### Isolation of Nuclei

J774A.1 cells were cultured on T75 culture flasks, as described above, 35 nM ACT was added and incubated with the cells at 37°C for 10 min. Cells were then washed three times with cold PBS, scraped and pelleted by centrifugation. All preparations were done at 4°C. The cells were gently resuspended in 500 µl hypotonic buffer (20 mM Tris-HCl, pH 7.4, 10 mM NaCl, 3 mM MgCl_2_,) and incubated on ice for 15 min. Then, IGEPAL® CA-630 (0.5% final concentration) was added and the resulting homogenate was centrifuged at 600 × *g* at 4°C for 10 min. The pellet, corresponding to the nuclear fraction, was resuspended in complete cell extraction buffer (100 mM Tris-HCl, pH 7.4, 2 mM Na_3_VO_4_, 100 mM NaCl, 1% Triton X-100, 1 mM EDTA, 10% glycerol, 1 mM EGTA, 0.1% SDS, 1 mM NaF, 0.5% deoxycholate, 20 mM Na_4_P_2_O_7_) for 30 min on ice with vortexing at 10 min intervals. Then, samples were centrifuged for 10 min at 14,000 × *g* at 4°C, and the supernatants (nuclear extracts) were aliquoted and stored at −80°C.

## Results

### ACT is Proteolytically Processed at its N-terminal AC domain by Cellular Calpain

**Figure 1:**
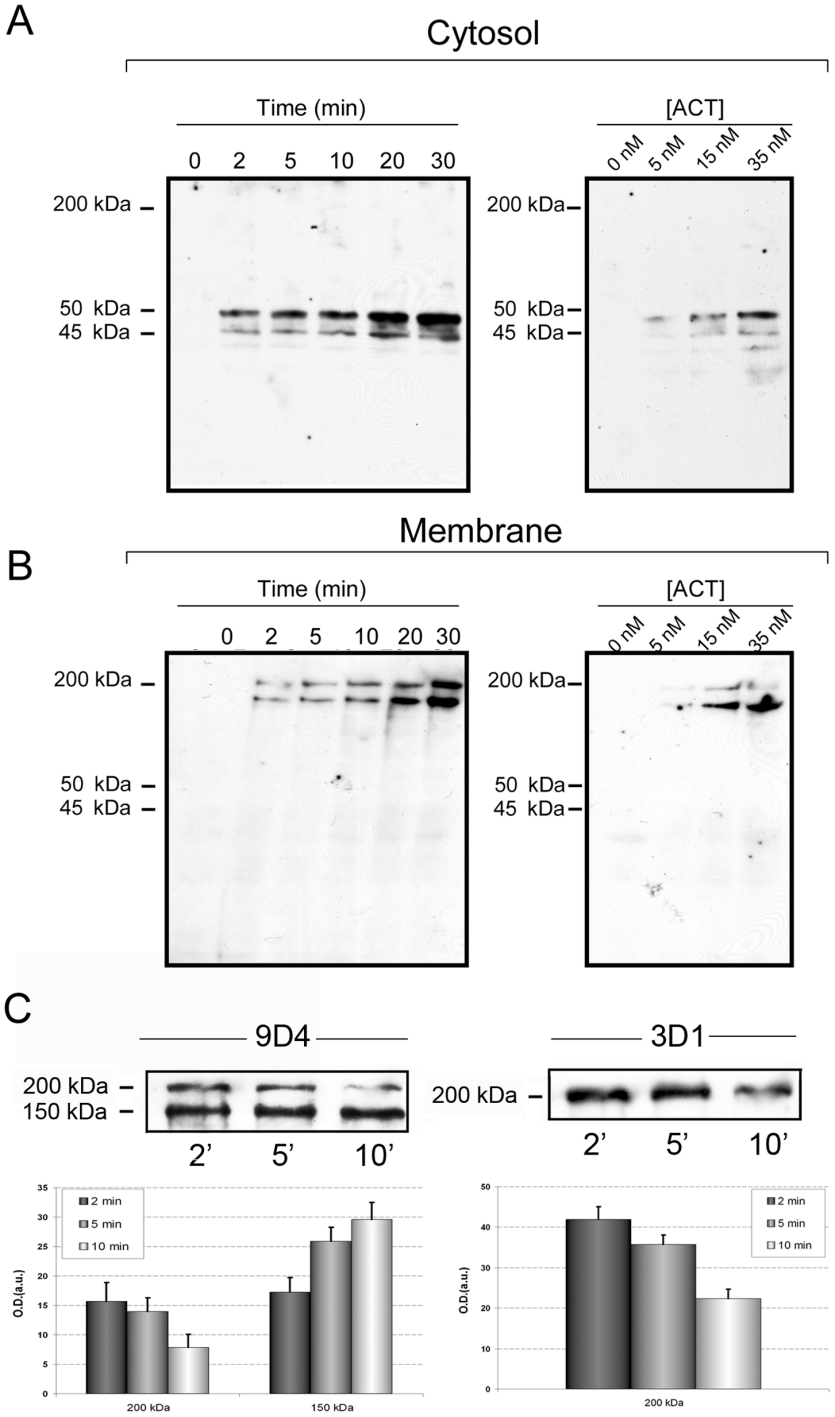
ACT is proteolytically cleaved in J774A.1 cells. Western blot analysis of cytosolic extracts (A) and membrane fractions (B) purified, as described in *Materials and Methods*, from J774A.1 cells treated with ACT (35 nM) at different incubation times (0–30 min) or different toxin concentrations (0–35 nM), and stained with anti-AC monoclonal antibody MAb 3D1 for the cytosolic fraction, and with MAb 9D4 for the membrane fraction. (C) Time course of the 200 and 150 kDa bands in membrane fractions of cells treated with 5 nM ACT at different incubation times. J774A.1 cells were incubated with ACT at 4°C and non-bound ACT was washed in ice-cold PBS as described in *Materials and Methods*. Then, cells were challenged at 37°C and the full length toxin and the 150 kDa fragment were determined in the membrane fractions of the cells with MAb 9D4 (left-hand panel) and the full length toxin was also detected by MAb 3D1 (right-hand panel). Densitometric quantification of the bands detected in the immunoblots is shown below the Western blots. A representative experiment from three independently performed assays is shown in (A) and (B), and a representative experiment from two independently performed assays is shown in (C).

J774A.1 macrophages exposed to the toxin at 37°C for different incubation times (0–30 min) and variable toxin concentrations, were lysed with hypotonic lysis buffer, and the cytosolic and membrane fractions separated and analyzed using two monoclonal antibodies, MAb 3D1 and MAb 9D4, which recognize epitopes at the AC and RTX domains, respectively [31]. Western blot analysis of the cytosolic fraction using MAb 3D1 revealed the presence of two protein bands with molecular masses of ≈ 45 and 50 kDa, whose intensity clearly increased with the time of treatment (**Fig. 1A** **, left-hand panel**). In parallel, analysis of the corresponding membrane fraction with MAb 9D4, revealed the presence of a protein band whose molecular mass corresponded to the entire protein (≈ 200 kDa) and a second band of ≈ 150 kDa, whose intensity increased with the time of treatment **(** **Fig. 1B** **, left-hand panel**). The masses of the protein bands showed good correlation with the apparent mass of the entire protein (≈ 200 kDa) and with RTX-fragments lacking the N-terminal end (≈ 150–160 kDa). **Figs. 1A and 1B** **, right-hand panels,** show the protein staining of cytosolic and membrane fractions, respectively, treated with variable toxin concentrations and revealed with 3D1 and 9D4 antibodies, respectively. A good correlation is observed between band intensities and toxin concentrations. The increment in the membrane fraction of both 200 kDa and 150 kDa bands with time and toxin concentration would be the consequence of the continuous ACT presence in the culture medium, thus not allowing a total clearance of the 200 kDa band. Therefore, and in order to demonstrate that the 200 kDa band is the source of the 150, 40 and 50 kDa bands, 5 nM ACT was added for 10 min to the cells at 4°C, allowing irreversible binding to the cells to better observe the 200 kDa band removal from the membrane. Then, the unbounded toxin was removed by several washes with ice-cold PBS, and the cells were further incubated at 37°C for different periods of time to follow the fate of the 200 kDa band. As shown in **Fig. 1C** **, left-hand panel**, under these conditions, the 200 kDa band decreased in a time-dependent manner, which in turn resulted in a reciprocal increment of a 150 kDa band, as detected by MAb 9D4. In parallel, the decrease of the 200 kDa band was also confirmed with MAb 3D1, **Fig. 1C** **, right-hand panel**, supporting the hypothesis that ACT cleavage yields the 40 and 50 kDa bands. A representative experiment from three independently performed assays under identical conditions has been chosen to appear in the figure. ACT treatment at the times and concentrations assayed does not give rise to a significant cell death as determined by LDH release assay (data not shown).

Altogether the data obtained strongly suggested that after cell incubation, ACT was proteolytically cleaved off into the macrophages and that, as a consequence of such processing MAb 3D1-positive AC domain fragments were released into the cell cytosol, whereas the MAb 9D4-positive C-terminal fragments of the toxin remained at the membrane.

**Figure 2:**
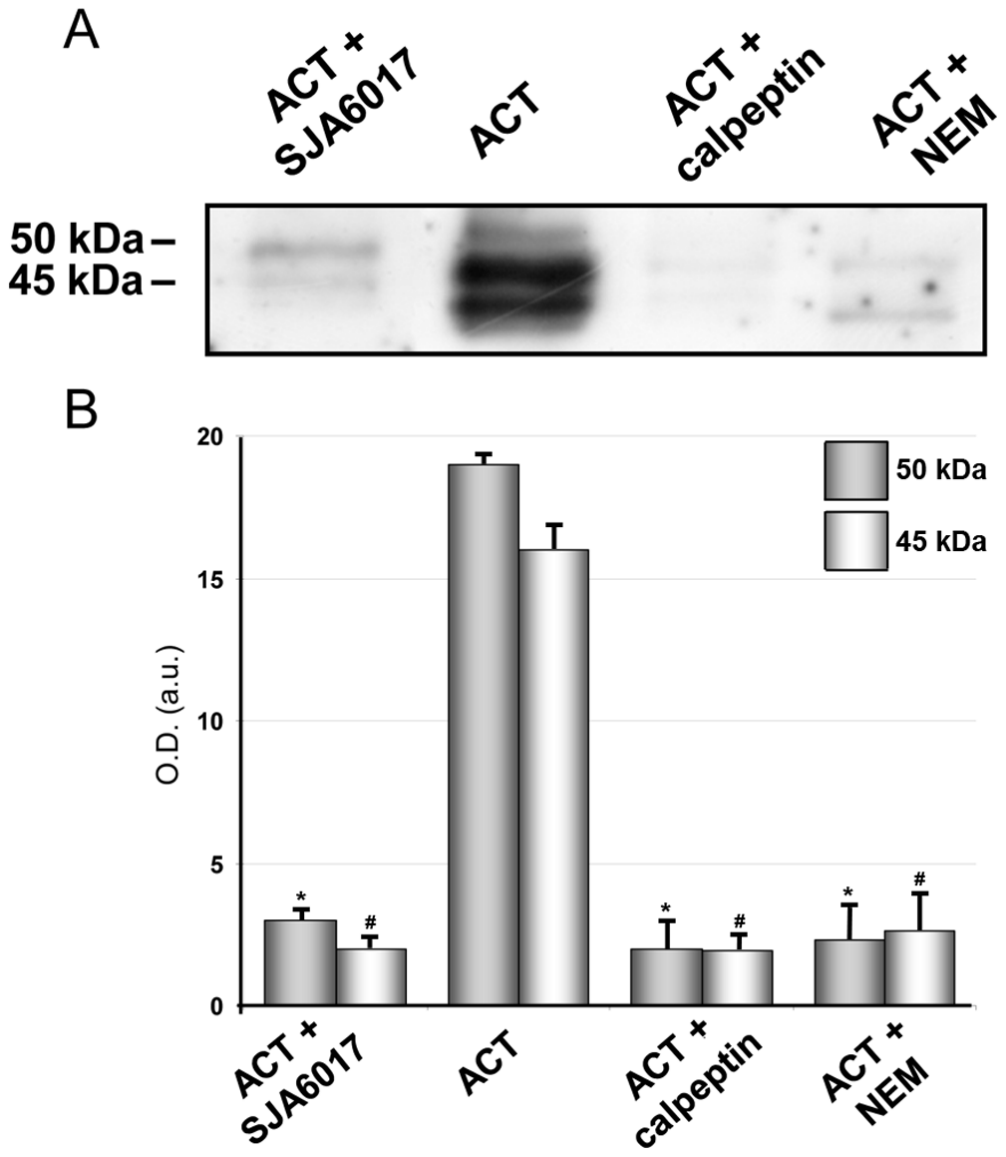
Calpain, the cellular cysteine protease responsible for ACT cleavage into J774A.1 cells. Effect of several inhibitors of cysteine proteases (100 µM calpeptin, 0.5 mM NEM and 78 nM SJA6017) on the proteolytic processing of ACT (35 nM) in macrophages, upon 10 minutes incubation of cells with the toxin, as assayed by Western blot of a cytosolic fraction purified from the ACT-treated cells. Cells were previously incubated with the various inhibitors for 30 min, before toxin addition (A) Densitometric quantification of the two major bands detected in the immunoblots (B). Levels of significance were determined by a two-tailed Student's t-test, and a confidence level of greater than 95% (p<0.05) was used to establish statistical significance. *p<0.001 with respect to 50 kDa band; #p<0.001 with respect to 45 kDa band.

ACT processing by an unknown N-ethylmaleimide-sensitive protease was previously reported in sheep erythrocytes [32]. N-ethylmaleimide has an inhibitory effect on cysteine proteases hence we checked the effect of various inhibitors of such a family of proteases on the ACT processing observed here in the macrophages. Three membrane permeable inhibitors were selected: N-ethylmaleimide (NEM), calpeptin and SJA6017. Before toxin addition, cells were pre-incubated for 30 minutes at 37°C with 100 µM calpeptin or 78 nM SJA6017, and to avoid cellular toxicity 0.5 mM NEM was added prior to ACT treatment. The three inhibitors were found to be effective, precluding with similar high efficacy the proteolytic cleavage of the toxin as judged by the nearly total disappearance of the two protein bands with molecular masses of ≈ 45 and 50 kDa corresponding to the N-terminal end of ACT (**Fig. 2** **A and B**). Data shown in **Fig. 2** correspond to one representative experiment from three independent assays performed under identical conditions. Intracellular cAMP production in 35 nM ACT-treated cells was 40,418±3,500 pmol cAMP/mg protein in the absence of calpeptin, and 38,680±4,058 pmol cAMP/mg protein in the presence of calpeptin. Calpeptin and SJA6017 have a demonstrated specificity for cellular calpains [33]–[36], a class of calcium-activated cysteine proteases. Calpain was thus a very likely potential candidate to be the protease involved in the ACT processing observed in the macrophages.

**Figure 3:**
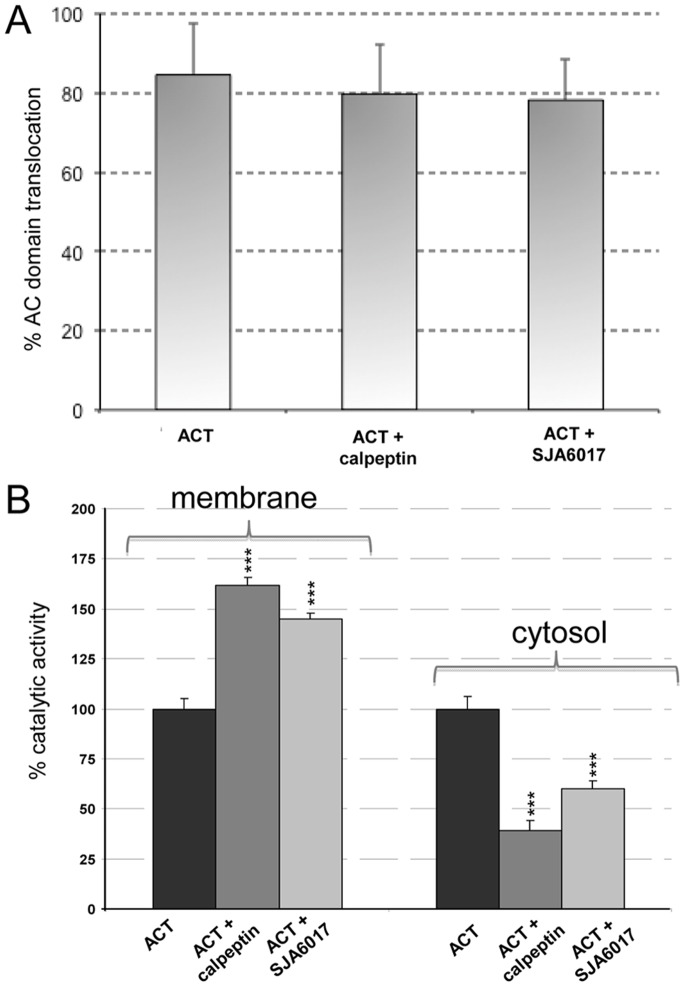
ACT catalytic domain translocation and cAMP production in membranes and cytosol in the presence of calpain inhibitors. J774A.1 cells were incubated for 10 min with 35 nM ACT in the presence or absence of calpain inhibitors (100 µM calpeptin and 78 nM SJA6017), translocation efficiency (A) and cAMP production in membrane and cytosolic fractions were determined as described in *Materials and Methods* (B). ***p<0.001 with respect to ACT in the absence of inhibitors.

We performed a control experiment to ensure that the lower amounts of N-terminal fragments detected in the cytosol upon treatment with the protease inhibitors were due to the blockage of the proteolytic cleavage by calpain and not to a blockage effect of these inhibitors on the translocation of the AC domain. Therefore we determined the percentage of translocation of the catalytic domain in macrophages treated with 35 nM ACT for 10 min, in the presence and absence of calpeptin and SJA6017, as described in *Materials and Methods* and it was found that AC domain translocation was very similar in the absence or presence of both inhibitors (**Fig. 3A**), and were similar to those described by Eby *et al.* [28]. We determined the enzymatic AC activity in membrane and cytosol fractions purified from the trypsinized ACT-treated cells, incubated in presence or absence of the inhibitors, as described in *Materials and Methods.* **Fig. 3B** shows that the cAMP production in the membrane fraction is higher in the presence of calpeptin or SJA6017, respectively 169±12 pmol cAMP/min×µg protein (control), 273±5 pmol cAMP/min×µg protein (calpeptin) or 233±35 pmol cAMP/min×µg protein (SJA6017). On the contrary, in the cytosolic fractions the cAMP production is higher in the absence of inhibitors, respectively 933±37 pmol cAMP/min×µg protein (control), 363±52 pmol cAMP/min×µg protein (calpeptin) and, 563±56 pmol cAMP/min×µg protein (SJA6017).

### “*In vitro*” Cleavage of Purified ACT by Calpain

**Figure 4:**
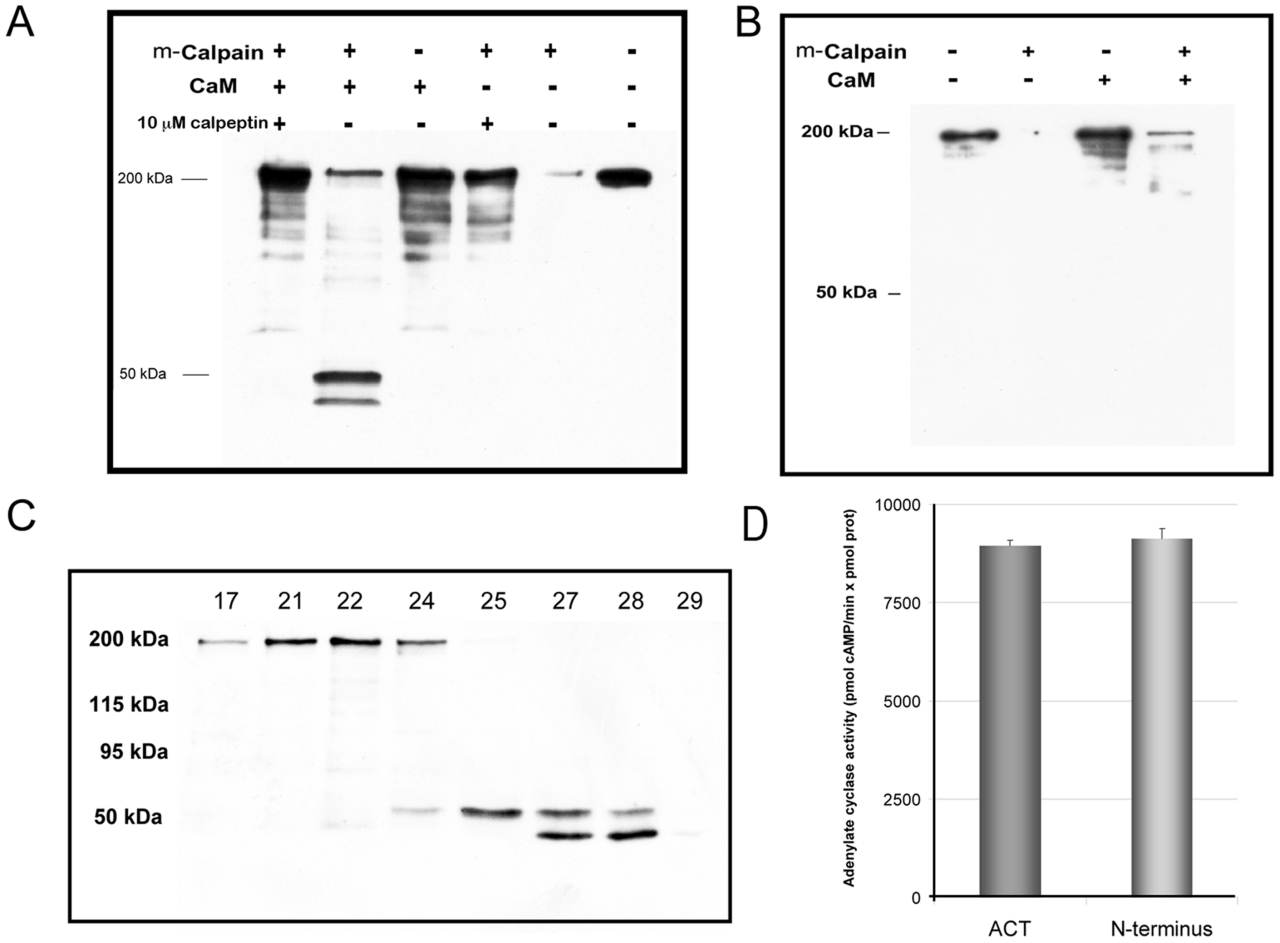
“*In vitro*” cleavage of purified ACT by calpain. Western blot analysis of an *in vitro* proteolysis assay in which purified ACT (5 µM) and calpain (100∶1 mol ratio) were incubated for 10 minutes at room temperature. Reactions were terminated by boiling the samples for 10 min in SDS-PAGE buffer. When indicated, calmodulin (10 µM) or calpeptin (10 µM) were included in the incubation buffer. MAb 3D1 was used to detect the N-terminus domain of the toxing after m-calpain cleavage. Results from one representative experiment out of three performed are shown in the figure (A). MAb 9D4 was used to detect the RTX domain of the toxin after m-calpain cleavage. Results from one representative experiment out of three performed are shown in the figure (B). The sample resulting from the proteolytic assay in the presence of calmodulin (corresponding to lane 2, from left to right, in the Western blot shown in panel A) was separated by molecular exclusion chromatography. The different fractions collected were analyzed by SDS-PAGE (8.5% polyacrylamide) and Western blot stained with the anti-AC monoclonal antibody MAb 3D1 (C). Catalytic activity of the intact purified ACT (1 nM) and of the fraction containing the two purified peptide bands (1 nM, final concentration with 2 nM calmodulin) was determined by ELISA as described in Methods section (D).

An *in vitro* cleavage assay was carried out to check whether the purified toxin is indeed a substrate of the protease calpain. Incubation of pure ACT (5 µM) with the protease (100∶1 ACT:calpain molar ratio) for 10 min at 37°C, induced a massive fragmentation of the N-terminal end, as judged by the absence of protein bands recognizable by the MAb 3D1 (**Fig. 4A**). These data suggested that the toxin N-terminal end might contain several potential cleavage sites for calpain, and that the AC domain might have different accessibility to the protease in the *in vivo* conditions with respect to the *in vitro* assay.

Upon translocation the AC domain binds to cellular calmodulin, which in turn, activates its enzymatic activity [13], [14]. To ascertain whether calmodulin binding may confer protection to the AC domain from massive N-terminal cleavage observed under *in vitro* conditions, a proteolysis assay was carried out in the presence of the toxin activator. The toxin (5 µM) was first pre-incubated with purified calmodulin (10 µM) at a 1∶2 ACT:CaM molar ratio (37°C, 10 min) and then purified calpain was added (1∶100 molar ratio, with respect to ACT). Two peptide bands, besides a fainter protein band of 200 kDa corresponding to the uncleaved ACT, were now visible after SDS-PAGE and western blot with MAb 3D1 antibody, (**Fig. 4A**). Inclusion of calpeptin (10 µM) in the assay precluded toxin cleavage (**Fig. 4A**). The molecular masses of the fragments detected in the presence of calmodulin were ≈ 45 and 50 kDa, very similar to the masses of the fragments detected in the *in vivo* assay (**Fig. 1A**). Data shown in **Fig. 4** correspond to one representative experiment from three independent assays performed under identical conditions. Nitrocellulose membranes were also probed against MAb 9D4 to detect the RTX domain and as shown in **Fig. 4B** two fragments of ≈ 200- and ≈ 150 kDa can be detected after calpain treatment of ACT. Data shown in **Fig. 4B** correspond to one representative experiment from three independent assays performed under identical conditions.

The peptides were separated from the uncleaved protein by molecular exclusion chromatography in a Superdex 200 10/300 GL High performance column (Tricorn Superdex) and their catalytic activity assayed as described under *Materials and Methods* (**Fig. 4C and 4D**, respectively). The peptides were catalyticaly active (fraction 27, containing an approximately equimolar fragment ratio, was tested for activity, 1 nM total concentration). For comparison, 1 nM ACT was also tested (**Fig. 4D**). Their cyclase activities were of 8,951±151 pmol cAMP/min×pmol protein for ACT and 9,117±211 pmol cAMP/min×pmol protein for the 45+50 kDa fragments. Mass spectrometry of tryptic fragments extracted from SDS-PAGE gels showed that both peptides corresponded to fragments derived only from the N-terminal end of the toxin. The 45 kDa band yielded tryptic peptides extending at least from residues 1 to 413 of ACT, while the 50 kDa band yielded peptides extending at least from residues 1 to 435. For the 45 kDa fragment, the score for the M1-R15 peptide was 65, and the confidence-threshold for a p<0.05 was 32. Peptide S414-R435 had a score of 8, and the Q400-R413 peptide had a score of 75. Thus it was estimated that this 45 kDa fragment contained at least the sequence from 1 to 413. For the 50 kDa fragment, the first and last peptides identified were identical to those identified in the 45 kDa fragment. In the 50 kDa sample the score of the M1-R15 peptide was 103, and that of the S414-R435 peptide was 158, thus we assumed that the 50 kDa fragment contained at least the residues from 1 to 435. From these and the previous data we concluded that ACT is indeed a substrate for calpain, both *in vivo* and *in vitro*. The data also suggested that binding of calmodulin to the AC domain may take place after AC domain translocation but before calpain activation and subsequent AC domain proteolytic processing.

### Modulation of the Activity of the 45 and 50 kDa Bands Derived from the in vitro Cleavage Assay

**Figure 5:**
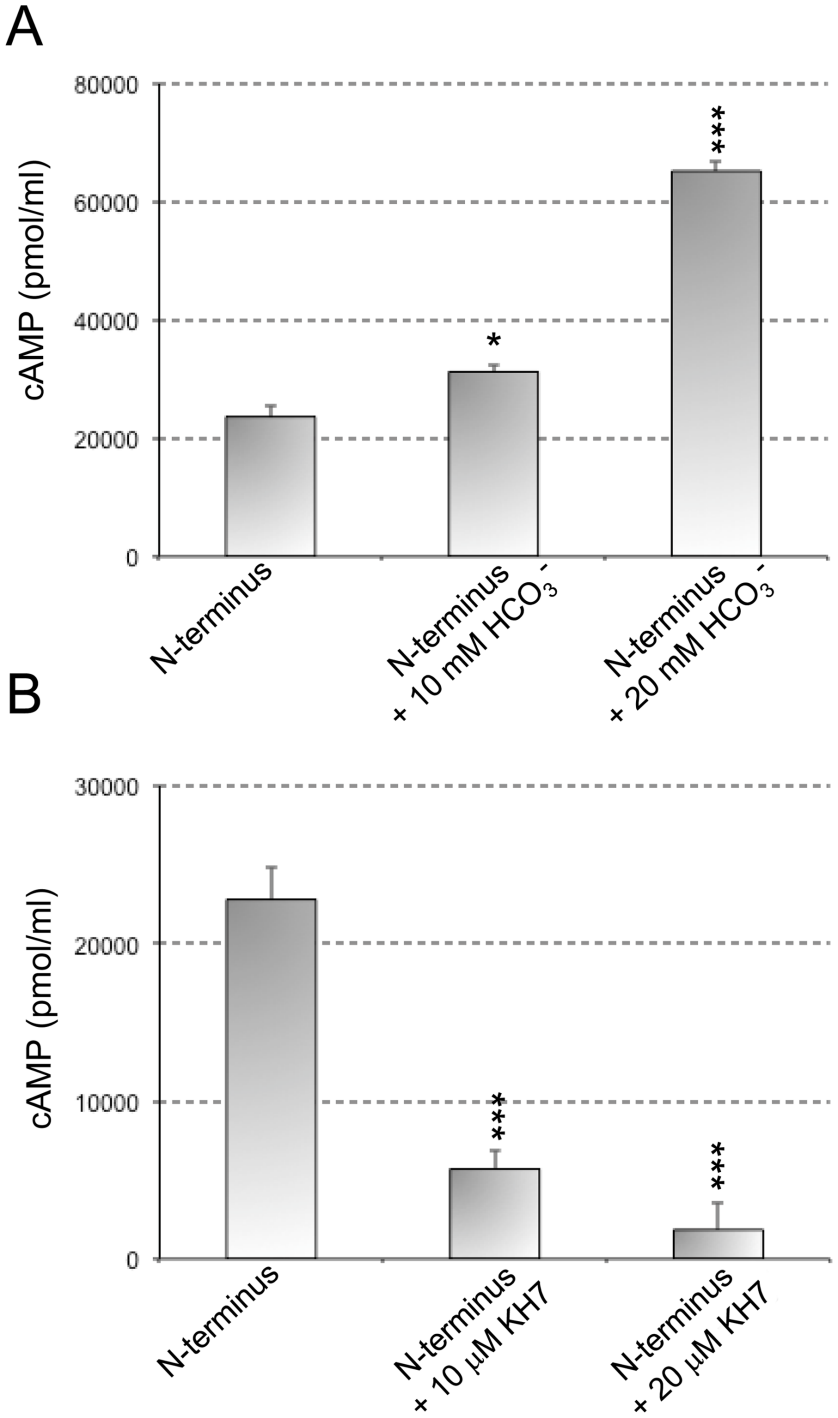
Effect of an activator and an inhibitor of soluble adenylate cyclases (sAC) on the enzyme activity of *in vitro* cleavage N-terminal fragments of ACT. The catalytic activity of N-terminal fragments purified from an *in vitro* ACT-cleavage assay was measured in presence of HCO_3_^-^ (activator of sAC) (A) and KH7 (inhibitor of sAC) (B) at two different concentrations. The cyclase activity was determined as described in *Materials and Methods*. *p<0,05; ***p<0,001 with respect to N-terminal fragments in the absence of the compounds.

Soluble adenylate cyclases (sAC) are a recently identified source of cAMP distinct from the more widely studied source of cAMP, the transmembrane adenylyl cyclases. Their activity is uniquely regulated by bicarbonate anions, and they are distributed throughout the cytoplasm and in cellular organelles. Therefore, using the 45 and 50 kDa bands recovered from the *in vitro* calpain-mediated cleavage, we next examined the effects of HCO_3_^-^ and KH7, a well know activator and inhibitor of sAC, respectively, on AC activity from the 45 and 50 kDa purified fragments. Incubation of 45 and 50 kDa fragments with HCO_3_^-^ resulted in a threefold increase in AC activity, while incubation with KH7 inhibited almost completely AC activity (**Fig. 5**). Thus after toxin cleavage the resulting N-terminal domain is modulated in a similar way than soluble adenylate cyclases.

### Calpain Activation and ACT Cleavage are Triggered by ACT-induced Ca^2+^ Influx

Calpains selectively cleave proteins in response to cellular calcium signals [37], [38] and are activated when the intracellular [Ca^2+^] is raised. Our group had previously demonstrated that ACT induces intracellular Ca^2+^ rises in macrophages by influx of this cation through Ca^2+^-channels [16]. This prompted us to explore the possible interrelation between the toxin-induced Ca^2+^ rise, and the observed calpain activation and ACT cleavage. We performed two different assays to address the hypothesis: first, we verified the activity of the protease in the ACT-treated macrophages, using a cell-permeable fluorogenic calpain specific FRET substrate, the peptide (DABCYL)-TPLK∼SPPPSPRE(EDANS)-RRRRRRR-NH2. The measurement of calpain activity is based on the increase of donor fluorescence due to cessation of FRET between the EDANS and DABCYL groups upon cleavage [30]. Second, we tested the effect on calpain activation, of two pharmacological inhibitors, previously found to be effective in blocking the toxin-induced calcium influx [16].

**Figure 6:**
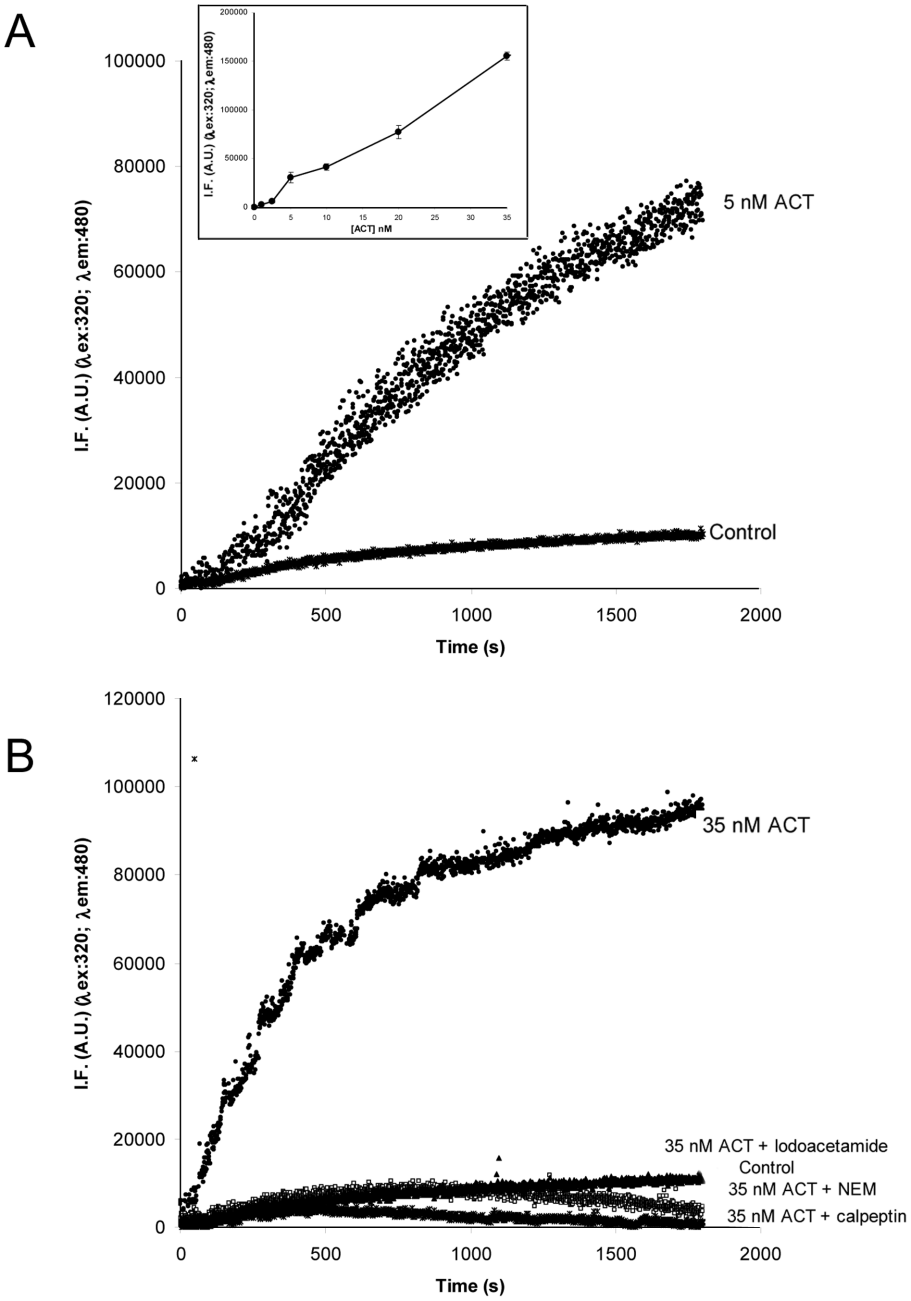
Fluorimetric calpain activity assay. Fluorimetric calpain activity assay using a cell permeable FRET-based substrate (DABCYL)-TPLK-SPPPSPRE(EDANS)RRRRRRR-NH2 (20 µM) incorporated into J774A.1 cells. Kinetics of the fluorescence signal of the fluorogenic substrate, recorded in control cells and cells treated with ACT (5 and 35 nM). The inset shows the toxin concentration dependence of the substrate fluorescence signal intensity. Fluorescence intensity values (IF) taken after 30 min cell incubation with the corresponding toxin amount are plotted. Data are the average ±SD value obtained from three independent experiments (A). Effect of cysteine protease inhibitors (100 µM calpeptin, 0.5 mM NEM and 10 µM α-iodoacetamide) on the proteolysis of the fluorogenic FRET-substrate in ACT-treated cells (B).

Toxin addition (5 nM) to cells loaded with the fluorogenic calpain substrate resulted in a rapid increase of fluorescence intensity as compared to untreated control cells (**Fig. 6A**). The increase in fluorescence was toxin-concentration dependent, and detectable for a broad range of ACT concentrations (**Fig. 6A** **, inset**). On the contrary, very low fluorescence signals, similar to the ones detected in control (untreated) cells, were recorded in the ACT-treated cells that had been preincubated with several formerly tested cysteine protease inhibitors, such as α-iodoacetamide (10 µM), NEM (0.5 mM) or calpeptin (100 µM) (**Fig. 6B**). These data show that in the ACT-treated cells calpain is activated, as judged by the productive cleavage on the fluorogenic calpain-specific substrate.

**Figure 7:**
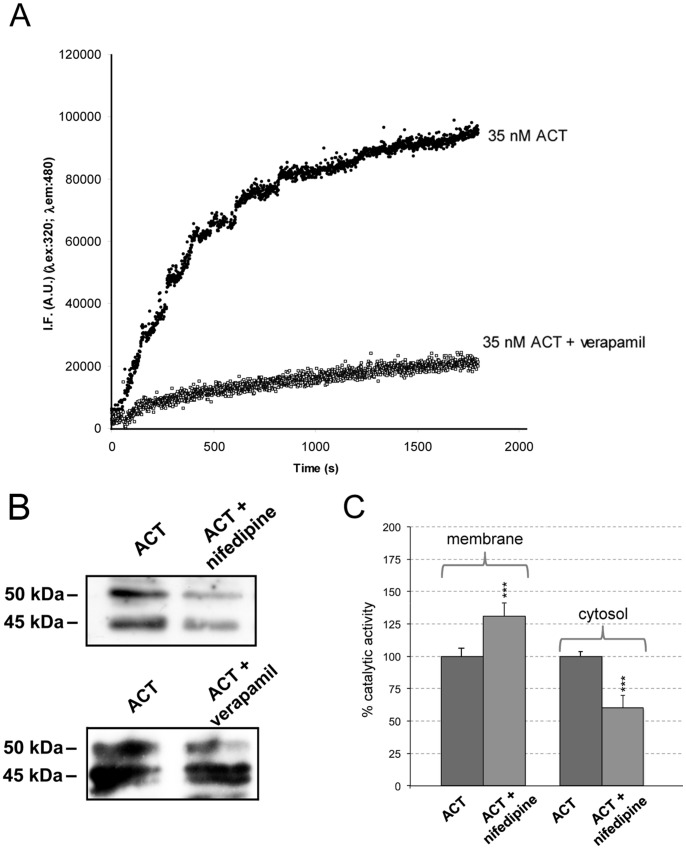
Calpain activation and ACT cleavage are triggered by the toxin-induced intracellular calcium increase. Effect of verapamil (100 µM), an antagonist of L-type calcium channels, on the kinetics of calpain-mediated proteolysis of the fluorogenic FRET-subtrate recorded in cells treated with ACT (35 nM) (A). Effect of the preincubation of cells with nifedipine (10 µM), and verapamil (100 µM), inhibitors of the L-type calcium channels, on the proteolytic cleavage of ACT in cells treated for 10 min with the toxin detected by Western blot (B). cAMP production in membrane and cytosolic fractions in the presence or absence of nifedipine (10 µM) (C). Assay conditions are described in *Materials and Methods section*. ***p<0,001 with respect to ACT in the absence of inhibitors.

We next addressed the possible interrelation between the toxin-induced Ca^2+^ rise, and the observed calpain activation and ACT cleavage. To this aim, verapamil (100 µM), a known antagonist of L-type Ca^2+^-channels and nifedipine (10 µM), a specific inhibitor of L-type Ca^2+^-channels, were tested. Nifedipine, which had been previously demonstrated to inhibit the calcium influx induced by ACT [16] could not be used with the fluorimetric calpain activity assay described above due to interferences with the fluorescence signal of the FRET substrate used in this assay. As depicted in **Fig. 7A and 7B**, respectively, both calpain activity and toxin cleavage, detected by Western blot, were greatly precluded by preincubation of the cells with the corresponding inhibitor (verapamil in the fluorimetric assay and both nifedipine and verapamil in the WB assay). These results also indicated that the calpain activation in ACT-treated macrophages is the consequence of the ACT-induced entry of extracellular calcium through L-type calcium channels, which fully agrees with previously reported data [**16**].

We also confirmed that AC domain translocation is not affected by nifedipine incubation and determined the cAMP production in the membrane and cytosol fractions of these samples. cAMP production in the membrane fraction is higher in the presence of nifedipine, 222±12 pmol cAMP/min×µg protein versus 169±12 pmol cAMP/min×µg protein in 35 nM ACT-treated cells. On the contrary, in the cytosolic fractions cAMP production is higher in the absence of the inhibitor, 933±37 pmol cAMP/min×µg protein, versus 567±18 pmol cAMP/min×µg protein in the presence of nifedipine. **Fig. 7C** **s**hows the higher cAMP production in the membrane fraction when nifedipine is present. On the contrary, in the cytosolic fractions cAMP production is higher in the absence of the L-type calcium channel inhibitor. These results support the previously proposed hypothesis that AC domain translocation takes place before toxin-mediated calcium influx [16].

**Figure 8:**
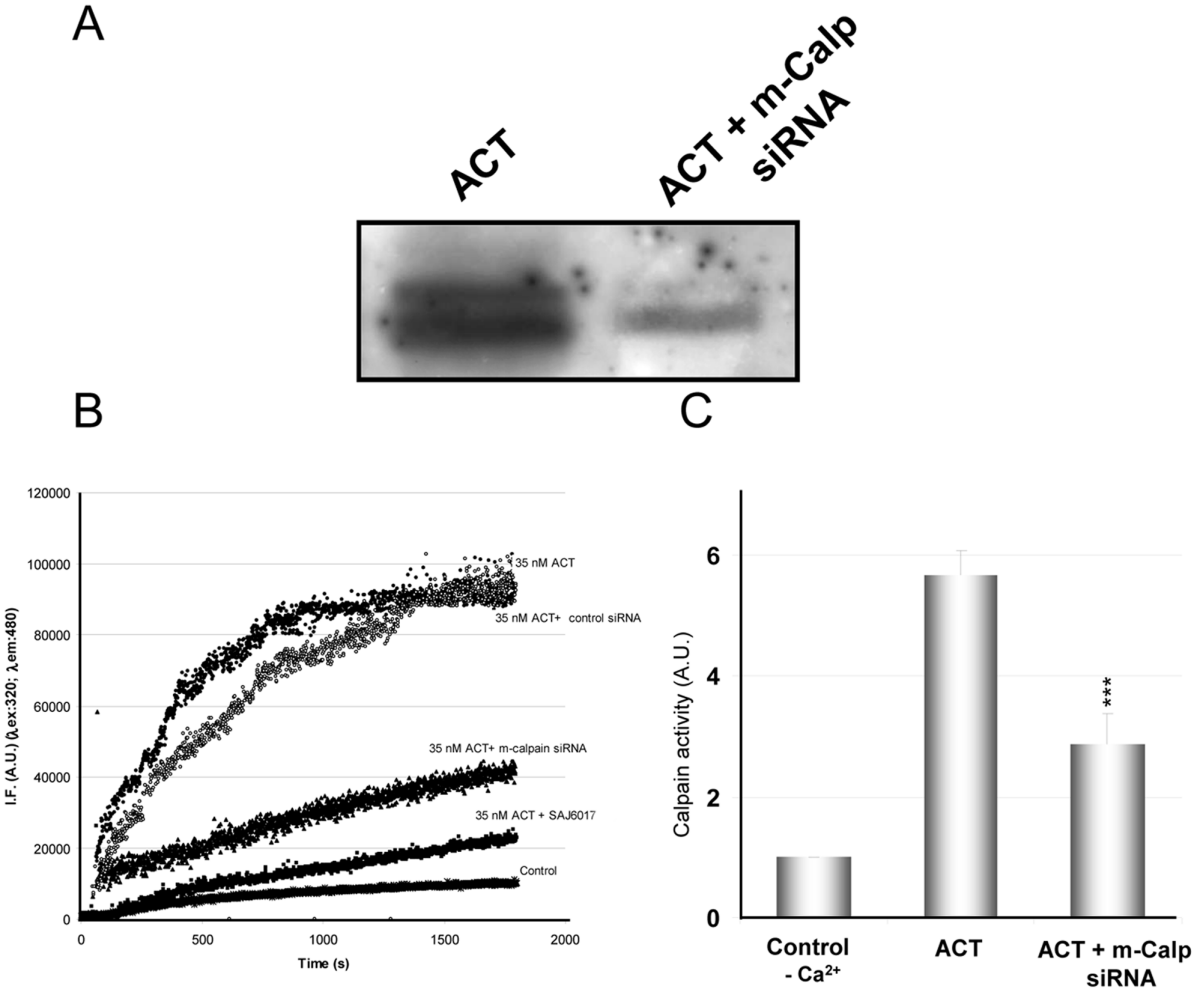
Activation of the m-calpain isoform in the ACT-treated cells. Western blot of the cytosolic fraction isolated from J774A.1 cells treated with ACT (35 nM) or from cells transfected with anti m-calpain siRNA and treated with ACT (35 nM) (A). Kinetics of the proteolytic cleavage of the FRET-based calpain specific substrate for ACT-treated cells (35 nM toxin), and cells in which m-calpain expression has been silenced by transfection with the corresponding siRNA, or mock cells transfected with scrambled control siRNA, or cells pre-incubated with SAJ6017 (78 nM), a specific m-calpain inhibitor. The figure is a representative experiment from three similar assays performed under identical conditions (B). Assay of calpain activity using FITC-labelled α-casein as substrate. FITC-α-casein hydrolysis by calpain is determined from in J774A.1 cell extracts incubated with FITC-labelled α-casein in the presence or absence of 1 mM CaCl_2_. Protease activity was quantified in 10% polyacrylamide gel by determining fluorescence intensity of the non-hydrolysed FITC-α-casein band. Non-digested FITC-α-casein and cell extracts without calcium were used as negative controls (C).

Finally, we performed a knock-down experiment with anti calpain siRNA (**Fig. 8**). Two major isoforms of calpain have been identified which differ according to the concentrations of Ca^2+^ required for their activation. The so-called μ-calpain requires micromolar levels of Ca^2+^ for activation whereas m-calpain requires millimolar levels [37], [38]. Expression of m-calpain was suppressed here by pretreatment of cells with a specific siRNA, as described in detail under *Materials and Methods*. The rationale behind silencing the m-calpain isoform is based on previous data of the calcium concentrations reached in cells treated with equivalent toxin doses [16], [17], which could be even higher locally. To analyze the effect of silencing the cellular m-calpain on the processing of ACT at its AC domain we performed a Western blot analysis of the cytosolic fractions isolated from transfected and non-transfected cells (**Fig. 8A**) and further densitometric analysis of the protein bands detected after staining with the monoclonal antibody 3D1. We determined, by densitometric quantitation, a decrease of about 46% in the appearance of the 50 kDa protein band and of about 37% in the 45 kDa band in the transfected cells as compared to control cells (**Fig. 8A**), thus suggesting that m-calpain is indeed directly involved in the proteolytic processing of ACT in the target cells. To determine the extent of the knock-down level achieved in the silencing experiment we used two functional assays, which are shown in **Fig. 8B** and **Fig. 8C**. In the first assay (**Fig. 8B** **)**, we used the fluorogenic calpain specific FRET substrate (see *Materials and Methods*). It is observed that the extent of cleavage of the fluorogenic substrate by calpain (which is activated upon addition of 35 nM ACT as in the assay shown in Fig. 6) was substantially lower in the transfected cells (trace 35 nM ACT+m-calpain siRNA) as compared to the non-transfected cells (trace 35 nM ACT) and similar to the cleavage obtained upon preincubation of the cells with the m-calpain specific inhibitor, SAJ6017 (78 nM). To verify the specificity of the anti m-calpain siRNA, control cells transfected with scrambled siRNA were also tested (control siRNA). In the second approach for siRNA knock-down efficiency quantification, we determined calpain activity using a FITC-labelled calpain substrate, FITC-α-casein (**Fig. 8C** **).** The figure shows the level of casein hydrolysis by calpain in ACT-treated cell lysates incubated with FITC-casein in the presence or absence of 1 mM CaCl_2_ and the hydrolysis level of this same fluorescent calpain substrate in cells transfected with the anti-calpain siRNA and incubated with 1 mM CaCl_2_. A significant 48% decrease in calpain activity on casein substrate in the siRNA transfected cells as compared to control non-transfected cells was observed. To further show the specificity of calpain for the casein substrate used in the assay, and its calcium dependence, the figure also shows the level of casein hydrolysis in cells preincubated in the absence of calcium. Data are the mean values ± SD of three independent experiments. Attempts to find whether the μ-calpain isoform is activated in the ACT-treated cells were also carried out, but the results obtained were less conclusive.

Altogether, these data reinforce our previous pharmacological and biochemical results, and indicate that in the ACT-treated cells (ACT 35 nM) the m-calpain isoform is clearly activated by the ACT-induced calcium influx, and is responsible for the proteolytic processing observed in the toxin, though involvement of the μ-calpain isoform cannot be ruled out, specially at lower toxin doses at which a lower calcium rise is expected to be achieved inside the target cell.

### Subcellular Localization of the AC Domain

**Figure 9:**
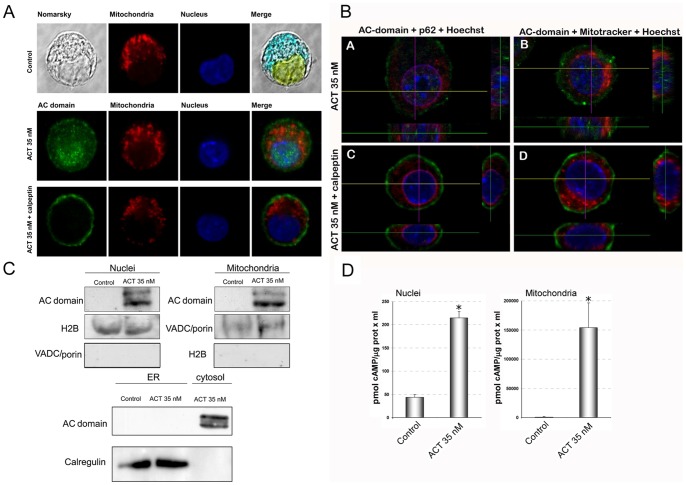
Upon cleavage by calpain, the “soluble” AC domain migrates to different subcellular organelles. Subcellular localization of the “soluble” AC domain as determined by confocal microscopy. Incubation of J774A.1 cells with ACT (35 nM) results in a colocalization of the “soluble” AC domain with the nucleus and the mitochodria after 10 min. Migration is prevented by pretreatment of cells with calpeptin (100 µM). Cells were preincubated for 30 min with the inhibitor prior to ACT addition, then incubated for 10 min with the toxin, washed three times, fixed and permeabilized to analyze AC domain by immunohistochemistry (A). Z-stack images obtained in the same conditions as in panel A, showing colocalization of “soluble” AC domain with the nucleus and mitochondria, and prevention of migration by pretreatment of cells with calpeptin (100 µM). Anti-nucleoporin p62 staining shows no colocalization of “soluble” AC domain with the nuclear envelope (B). Detection of the “soluble” AC domain into isolated nuclei, mitochondria or ER by Western blot analysis. Nuclear, ER and mitochondrial fractions isolated from cells treated with ACT (35 nM) for 10 min were analyzed by Western blot and stained with the anti-AC MAb 3D1. Anti-histone H2B antibody and anti-VDAC/porin antibody were used to stain histone H2B and the VDAC/porin as molecular markers of the nuclei and mitochondria, respectively. Calregulin was used as marker for ER fractions (C). Adenylate cyclase activity is enhanced very significantly both in nuclear extracts and mitochondrial fractions obtained from 35 nM ACT treated cells. cAMP production was determined as described in *Materials and Methods* (D).

The intracellular fate of the released AC domain was examined by confocal microscopy after staining with MAb 3D1. As documented by representative images shown in **Fig. 9A**, taken from about 30 cells, from one of three independent experiments performed, 10 minutes after the cell treatment with ACT (35 nM), most of the AC fluorescence signal (green) showed a punctate distribution that colocalized with the cell nucleus (45±10% colocalization, image analysis was performed using a macro in WCIF Image J software). ACT presence was also detected close to, or surrounding, the mitochondria, though in this case the colocalization values were not so conclusive (23±8%). A faint staining at the plasma membrane was still visible after this incubation time (**Fig. 9A**). These results suggested that after ACT cleavage, the released “soluble” AC domain migrates intracellularly to different organelles, nucleus and probably mitochondria.

In the cells that had been pre-incubated with calpeptin before toxin treatment, the green fluorescence signal corresponding to the AC domain was only visible at the cell plasma membrane after 10 minutes of ACT treatment, with no detectable subcellular fluorescence signal (**Fig. 9A**). This is in agreement with the previous result shown in **Figs. 2** and **6** that calpeptin efficiently inhibits toxin cleavage by calpain, and with the notion that by inhibiting toxin cleavage the subcellular migration of the “soluble” AC domain is prevented. We further confirmed these results by acquiring Z-stack images in the confocal laser-scanning microscope, which also showed a nuclear localization of the AC domain (**Fig. 9B**). Colocalization of AC domain with the nuclear envelope was discarded because there was no colocalization with nucleoporin (**Fig. 9B**). As shown in **Fig. 9B**, localization of AC domain in the nucleus was precluded with the calpeptin treatment.

To further confirm the specific nature of this subcellular localization for the “soluble” AC a Western blot analysis was performed with nuclei, mitochondria and ER preparations isolated from the ACT-treated cells (35 nM ACT, 10 min incubation at 37°C). As documented by a representative blot shown in **Fig. 9C**, staining of the AC domain with MAb 3D1 was visible both in nuclear fractions and in mitochondrial preparations isolated from the cells after ACT treatment. As a control, cross-reactivity of H2B histone in the mitochondrial preparation and of the VDAC/porin in the nuclei fraction was assayed and discarded. Additionally, an ER-enriched subcellular preparation was also tested with 3D1. As shown in the figure, the results obtained were negative (**Fig. 9C**).

To test whether the *in vivo* cleavage event was functionally relevant, we assayed next adenylate cyclase activity in the nuclear and mitochondrial fractions isolated from 35 nM ACT-treated cells (non treated cells were used as control). cAMP production was assayed as described under *Materials and Methods*. As shown in **Fig. 9D**, nuclear extracts and mitochondrial enriched fractions presented a very significant increment in adenylate cyclase activity, confirming the premise that after calpain cleavage, the AC domain which migrates to different compartments retains its catalityc activity. This cleavage event would confer to ACT the capacity of a coordinated production of different cAMP “pools” in different cellular compartments.

## Discussion

We report here on two novel and relevant findings: first we demonstrate that after translocation ACT is cleaved off inside the phagocytes at its N-terminal domain by calcium-activated cellular calpain, and second, that the released enzymatically active “soluble” AC domain migrates intracellularly to colocalize to the nucleus and mitochondria. The proteolytic processing of a bacterial toxin by host calpains represents a unique mechanism that confers to ACT the possibility to generate the important second messenger cAMP either underneath the plasma membrane or in various subcellular organelles.

We show evidence that ACT is a novel substrate, both *in vivo* and *in vitro*, for the neutral cysteine protease calpain. Previously described calpain substrates were of eukaryotic origin. Calpain is not a lysosomal protease, but it plays a regulatory processing role in eukaryotic cells, rather than a degradative function. No specific amino acid sequence is uniquely recognized by calpains [37], [38]. Amongst protein substrates tertiary structure elements rather than primary amino acid sequences are likely responsible for directing cleavage to a specific substrate. We identified upon *in vitro* cleavage of purified ACT two catalytically active peptides with masses of ≈45 and 50 kDa, whose proteomic analysis by tryptic fragmentation revealed that such fragments contain at least peptides from residues 1 to 413 and 1 to 435 of the ACT polypeptide chain, respectively. We have identified by computational analysis [39] three potential calpain cleavage-sites, residues 432, 464 and 491, which would explain the appearance of two catalytic bands. These sites are attractive potential mutation candidates to be explored in a future investigation.

Calpains selectively cleave proteins in response to cellular calcium signals [37], [38]. Under physiological conditions, a transient and localized calcium influx into the cell activates a small local population of calpains, which then advance the signal transduction pathway by catalyzing the controlled proteolysis of its target proteins [37], [38]. We demonstrate here that in the ACT-treated macrophages calpain is activated by the rise in intracellular [Ca^2+^] triggered by the toxin. Both calpain activation and subsequent ACT processing by the protease are importantly precluded by preincubation of cells with specific inhibitors of L-type calcium channels (nifedipine and verapamil), indicating that the influx of extracellular calcium involved in protease activation takes place through L-type calcium channels, confirming previous findings by our group [16]. In addition, the efficient AC domain translocation detected in the presence of calpain inhibitors and of L-type calcium channels supports the hypothesis that AC translocation precedes the rise in the intracellular [Ca^2+^] triggered by ACT [16]. However we cannot exclude that a small increment in cytosolic [Ca^2+^] would take place by the suggested ACT-induced extracellular cation entry through a toxin “intermediate” participating itself in formation of a transiently opened Ca^2+^-conducting path across the cell membrane [17], [40]. Our silencing and pharmacological inhibition assays suggest that the so-called m-calpain isoform is mainly activated in the ACT-treated macrophages at the toxin doses tested (35 nM), though it is possible that at lower toxin concentrations, thus lower calcium increases triggered by ACT, only the μ-calpain isoform is activated.

We observe here, in the *in vitro* proteolysis assay, that toxin preincubation with calmodulin fully protects the toxin N-terminal domain from a massive proteolysis by calpain, which suggests that calmodulin might play a role as structural stabilizer of the AC domain into the cell, besides its known role as enzymatic activator. Several observations by other authors are consistent with this notion. Ladant found that calmodulin binding to the toxin increased considerably the resistance of adenylate cyclase to inactivation by trypsin [41]. More recently, it was shown that a purified catalytic domain corresponding to residues 1–384 of ACT is thermodynamically not very stable and prone to aggregation in a temperature-dependent manner, while in presence of calmodulin the complex undergoes a significant compaction and dehydration [42]. The binding of calmodulin to the AC domain would provide in fact a structural stabilization and/or a shielding of the AC domain to circumvent premature or inappropriate cleavage by the active protease, which would ensure the release of an active and full AC domain.

Finally, we show that upon cleavage at the N-terminal domain, the “soluble” AC domain is localized inside the nucleus and with mitochondria. Detection of a “soluble AC” domain in the nucleus and mitochondria of the ACT-treated phagocytes, and the fact that the ACT N-terminal region covering the full catalytic domain (aminoacids 1–384) does not display any membrane association propensity, immediately raises the supposition of a potential requirement of a targeting sequence or region in the “soluble AC form” to favor its migration to the subcellular organelles. Interestingly, we determined by mass spectrometry of tryptic fragments of the two protein fragments (45 and 50 kDa) derived from calpain *in vitro* cleavage that both peptides extend beyond aminoacid 384, to residue 413 in the case of the 45 kDa fragment, and to aminoacid 435 in the case of the 50 kDa fragment. Very recently Karst et al. [43] have reported that the region 375–485 of ACT appears to have an important role in promoting the interaction of the catalytic domain with the plasma membrane and its translocation across the lipid bilayer of target cells. These data lead us to speculate that the proteolytic cleavage of ACT by calpain might precisely release an ACT “soluble AC” form endowed with both catalytic capacity and ability to ensure a specific subcellular localization and cAMP production.

ACT is hypothesized to have originated from a gene fusion between an ancestor of the RTX family and a eukaryotic calmodulin-regulated adenylate cyclase [10]. Recently, the presence of “soluble” cytosolic adenylyl cyclases (sAC) was discovered in mammalian cells, evolutionarily, structurally and biochemically distinct from the G-protein-responsive transmembrane adenylyl cyclases [44]. These sAC are associated with various intracellular organelle, including mitochondria, centrioles, mitotic spindle and nuclei [45] where they stimulate several cAMP-dependent signaling pathways such as CREB phosphorylation [46] or mitochondrial-dependent apoptosis in endothelial cells [47]. In view of our present findings it is tempting to speculate that the enzymatic component of ACT could come from such a “soluble adenylyl cyclase” form. This idea is supported by the finding that, in the presence of HCO_3_^-^, a well know activator of sAC [46], the activity of the 45 and 50 kDa fragments derived from calpain *in vitro* cleavage is significantly enhanced, and in the presence of the specific sAC blocker, KH7 [47], the activity of those fragments is significantly inhibited (**Fig. 5**).

In conclusion, our study proposes a new scenario in the toxin mechanism of action: we show that after AC domain translocation, the toxin induced calcium influx promotes calpain activation. The calpain-mediated ACT processing would confer to the toxin the capacity of a spatially and temporally coordinated production of different cAMP “pools”, which would play different roles in the cell pathophysiology.
